# GeneNotes – A novel information management software for biologists

**DOI:** 10.1186/1471-2105-6-20

**Published:** 2005-02-01

**Authors:** Pengyu Hong, Wing H Wong

**Affiliations:** 1 BioX program, Department of Statistics and HRP, Stanford University, Stanford, CA 94305-4065, USA; 2 BioX program, Department of Statistics and HRP, Stanford University, Stanford, CA 94305-4065, USA

## Abstract

**Background:**

Collecting and managing information is a challenging task in a genome-wide profiling research project. Most databases and online computational tools require a direct human involvement. Information and computational results are presented in various multimedia formats (e.g., text, image, PDF, word files, etc.), many of which cannot be automatically processed by computers in biologically meaningful ways. In addition, the quality of computational results is far from perfect and requires nontrivial manual examination. The timely selection, integration and interpretation of heterogeneous biological information still heavily rely on the sensibility of biologists. Biologists often feel overwhelmed by the huge amount of and the great diversity of distributed heterogeneous biological information.

**Description:**

We developed an information management application called GeneNotes. GeneNotes is the first application that allows users to collect and manage multimedia biological information about genes/ESTs. GeneNotes provides an integrated environment for users to surf the Internet, collect notes for genes/ESTs, and retrieve notes. GeneNotes is supported by a server that integrates gene annotations from many major databases (e.g., HGNC, MGI, etc.). GeneNotes uses the integrated gene annotations to (a) identify genes given various types of gene IDs (e.g., RefSeq ID, GenBank ID, etc.), and (b) provide quick views of genes. GeneNotes is free for academic usage. The program and the tutorials are available at: .

**Conclusions:**

GeneNotes provides a novel human-computer interface to assist researchers to collect and manage biological information. It also provides a platform for studying how users behave when they manipulate biological information. The results of such study can lead to innovation of more intelligent human-computer interfaces that greatly shorten the cycle of biology research.

## Background

In a genome-wide profiling project, researchers usually select many sets of genes (or ESTs) for further investigation based on the computational analyses of the experimental data. For example, a set of genes is selected because they are clustered together based on their mRNA levels. To generate or support biologically meaningful hypotheses, researchers need to selectively and systematically collect information about the genes/ESTs from various sources. For example, huge amount of gene annotations have been accumulated over decades in distributed databases. In addition, there are plenty of online computational tools (e.g., BLAST [1], TMHMM [2], MEME [3], etc.) that can be customized to generate invaluable information complementary to the local computational analyses of researchers. Most databases and online computational tools require a direct human involvement. Sometimes, it is extremely difficult, if not impossible, to perform further computational analyses on the collected information. For example, a cellular image often contains large amount of important information that can be easily understood by human but is beyond the capability of existing computational tools. Therefore, the timely integration and interpretation of the collected information still relies heavily on the sensibility of biologists. However, it is a challenging task to effectively collect and manage various types of information of interest. Researchers often feel overwhelmed by not only vast amount of information but also the great diversity of information types. To facilitate this task, we developed GeneNotes that provides an integrated environment for surfing the Internet, recording information, annotating genes, and retrieving information in the form of text, HTML, image, PDF, word file, and so on.

## Implementation

### Architecture

**Figure 1:**
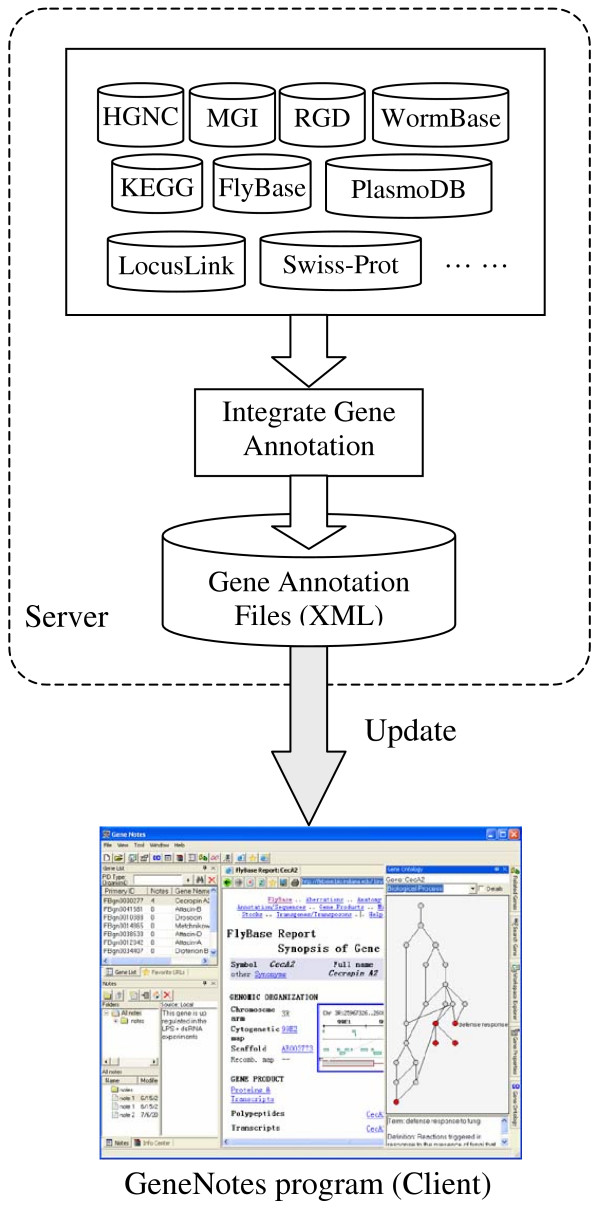
The server integrates gene annotations from many major biological databases.

We maintain a server (Figure 1) that integrates gene annotations from many major databases (e.g., HGNC [4], MGI [5], RGD [6], FlyBase [7], WormBase [8], PlasmoDB [9], SGD [10], KEGG pathway DB [11], etc.). The integrated gene annotations are stored as XML files, which are updated every two weeks. GeneNotes runs locally on users' computers and uses the integrated gene annotations to identify genes given various types of IDs (e.g., RefSeq ID, GenBank ID, UniGene ID, LocusLink ID, etc.). There is one menu in GeneNotes that allows the user to update the annotation files from the server. Once a gene is identified, GeneNotes can visualize Gene Ontology annotation of the gene, list its other annotations (e.g., name/symbol/synonyms/products, RefSeq ID, GenBank ID, UniGene ID, LocusLink ID, Model Organism database ID, protein information, etc.), and generate hyperlinks to major databases (e.g., Swiss-Prot [12], UCSC Genome DB [13], KEGG pathway DB [11], etc.) for the user to jump around those databases easily. GeneNotes also supports Affymetrix probe sets given the corresponding GeneChip^® ^annotation files, which can be downloaded from the web site of Affymetrix.

**Figure 2:**
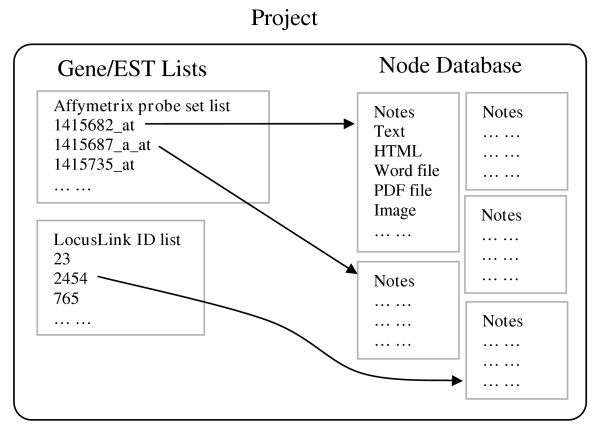
GeneNotes organizes information by projects.

GeneNotes manages information by projects (Figure 2). Each project contains a note database and several lists of genes/ESTs. The gene/EST lists are selected by users and can be edited anytime via the GUI of GeneNotes. Each gene/EST can have many notes that contain multimedia information. For example, a note could be a segment of text, an image, a PDF file, a word file, a local copy of a web page, and so on.

### Create, collect, and manage notes

GeneNotes offers many useful functions. The most attractive feature to biologists is the function for creating, collecting, and managing notes for genes/ESTs. Basically, there are three ways for creating/collecting notes. First, GeneNotes has an embedded Web browser, which allows the user to surf the Internet. The user can selectively record useful information segments shown in the Web browser or save a web page as a note. Saving a web page as a note is especially useful when the web page contains the non-text results (e.g., images, logos, etc.) generated by a remote computational server. GeneNotes automatically stores the sources of notes and allows users to trace back to the sources of notes later. Second, users can create text notes to summarize their thoughts/comments about genes/ESTs. Finally, users can create notes linked to local files (e.g., images, PDF files, word files, etc.). The user can directly modify a text note and add annotations to non-text notes. Each note has an editable title. A meaningful title helps the user recall what information the note contains. When the number of notes is large, it may become difficult for users to browse and locate notes. GeneNotes allows users to organize notes into directories, which can be defined by categories.

### Note database

Currently, GeneNotes runs on Microsoft Windows 2000/XP and uses Microsoft Data Access Components (MDAC) 2.7 to manipulate the note database. MDAC 2.7 provides the same Data Access core components as Windows XP SP1. Windows 2000 users need to download and install MDAC 2.7 or the latest version of MDAC, which are free at the web site of Microsoft. We are considering developing a platform independent version of GeneNotes and using other free database software such as MySQL [14].

### GUI

The GUI of GeneNotes is flexible and user friendly. It contains the following main windows: project explorer, gene list window, note window, gene property window, Gene Ontology visualization window, and web browser window. The project explorer displays the content of a project, e.g., gene lists, the note database file, Affymetrix GeneChip^® ^annotation files, etc. It allows users to edit the project, e.g., add/delete gene lists, create/change the note database, add/delete Affymetrix GeneChip^® ^annotation files, etc. The user can select a gene list in the project explorer. The selected gene list will be displayed in the gene list window. When a gene is selected in the gene list window, its properties (e.g., name/symbol/synonyms, RefSeq IDs, GenBank IDs, UniGene ID, etc.) will be displayed in the gene property window and its Gene Ontology annotation will be visualized in the Gene Ontology visualization window. The gene property window also displays a set of hyperlinks that are linked to the records of the selected gene in remote biological databases (e.g., HGNC [4], MGI [5], RGD [6], FlyBase [7], WormBase [8], PlasmoDB [9], SGD [10], Swiss-Prot [12], UCSC Genome DB [13], KEGG pathway DB [11], etc.). Once a hyperlink is clicked, GeneNotes will display the corresponding URL in the web browser window. The web browser window allows user to surf the Internet freely.

All windows are dockable. Users can configure of the interface by dragging and dropping the window to virtually wherever they want. If a window is set as auto-hide, it will hide itself when it is inactive so that the computer screen can be used to display other windows.

## Discussion and conclusions

GeneNotes provides a novel human-computer interface for researchers to effectively and efficiently manage multimedia biological knowledge. There are many online databases (e.g., Entrez Gene [15], GeneCards [16], etc.) providing pre-compiled information about genes/ESTs. GeneNotes has different purposes. It gives users freedom to selectively collect information and create their own databases to store heterogeneous biological information. At the same time, GeneNotes allows users to make full usage of those online databases. GeneNotes stores notes locally so that the notes are accessible when computers are offline. GeneNotes organizes information in the order of project, gene/EST list, and notes. This not only makes it easier for researchers to switch from one gene/EST to another one but also helps researchers obtain global view about the ongoing status of the project. We are currently pursuing the direction to innovate more intelligent human-computer interface to help biologists collect and manage information by observing how biologists using GeneNotes.

## Availability and requirements

Project name: GeneNotes

Project home page: .

Operating system(s): Windows 2000/XP

Programming language: C#

License: Free for non-commercial usage.

## Authors' contributions

PH was responsible for the conception, design, implementation, and testing of the GeneNotes program. WHW was responsible for its conception, design, and testing and provided overall project coordination. Both authors have read and approved the final manuscript.
